# Intrinsic Motoneuron Excitability Differentiates Sarcopenic, Nonsarcopenic and Athletic Ageing Phenotypes

**DOI:** 10.1002/jcsm.70126

**Published:** 2025-11-25

**Authors:** Lucas B. R. Orssatto, David Scott, Brian C. Clark, Jeffrey Lim, Robin M. Daly

**Affiliations:** ^1^ Centre for Sensorimotor Performance, School of Human Movement and Nutrition Sciences The University of Queensland Brisbane Queensland Australia; ^2^ Institute for Physical Activity and Nutrition, School of Exercise and Nutrition Sciences Deakin University Burwood Victoria Australia; ^3^ Department of Medicine, School of Clinical Sciences at Monash Health Monash University Clayton Victoria Australia; ^4^ Ohio Musculoskeletal and Neurological Institute (OMNI) Ohio University Athens Ohio USA; ^5^ Department of Biomedical Sciences, Heritage College of Osteopathic Medicine Ohio University Athens Ohio USA

**Keywords:** dynapenia, functional capacity, HD‐EMG, motor unit, muscle, persistent inward currents, sex ageing

## Abstract

**Background:**

The mechanisms underlying sarcopenia‐related physical decline remain poorly understood, particularly with respect to neural contributions. Muscle atrophy has traditionally been viewed as the primary driver, but growing evidence suggests that neuromuscular impairments—especially reduced intrinsic motoneuron excitability—may play a central role. This intrinsic excitability, which is critical for modulating motoneuron discharge rates, likely contributes to age‐related weakness and mobility loss. We investigated whether intrinsic motoneuron excitability differs across older adults with sarcopenia, nonsarcopenic controls and masters athletes and whether these differences relate to physical function.

**Methods:**

Fifty‐six older adults (74.3 ± 7.2 years, 50% female), including 12 sarcopenic, 23 nonsarcopenic controls and 21 masters athletes, were recruited. The Sarcopenia Definitions and Outcomes Consortium (SDOC) thresholds were used for sarcopenia screening. High‐density electromyography (HD‐EMG) was recorded from the tibialis anterior during ramped isometric contractions at intensities of 20%, 40% and 60% of maximum torque (i20%, i40%, i60%). A total of 4998 decomposed motor units were categorized by recruitment thresholds (rt0%–20%, rt20%–40% and rt40%–60%). Paired motor unit analysis was used to calculate delta frequency (Δ*F*), an established index of intrinsic motoneuron excitability primarily reflecting persistent inward currents (PICs) contribution to discharge behaviour. Muscle strength, power and physical function were assessed using established performance‐based tests.

**Results:**

Sarcopenic older adults had significantly lower dorsiflexion peak torque (−56%), sit‐to‐stand power (−37%) and functional capacity tests performance (−30 to −46%) compared to controls. Master athletes demonstrated higher sit‐to‐stand power (23%) and functional performance (11% to 23%) than controls. Δ*F* was significantly lower in sarcopenic individuals compared to both controls and master athletes across all contraction intensities and recruitment threshold bins (−22% to −38%). Master athletes did not differ from controls in Δ*F* for low‐threshold units (rt0%–20%) or at i20% and i40% contraction intensities. However, Δ*F* was higher in athletes than controls at i60% for mid‐ and high‐threshold units (rt20%–40% and rt40%–60%) by 15% and 20%. These group differences in Δ*F*, particularly at higher intensities, were associated with the degree of muscle weakness and physical limitations.

**Conclusions:**

Intrinsic motoneuron excitability, as estimated by Δ*F*, is substantially reduced in this group of sarcopenic older adults, suggesting that it may be critical to functional capacity. Long‐term exercise practice preserves excitability, particularly during high‐demand motor tasks. These findings identify intrinsic motoneuron excitability as both a mechanistic marker of neuromuscular ageing and a potential target for investigations of novel interventions aiming to restore neuromotor function in sarcopenia.

## 1 Introduction

The mechanisms underlying sarcopenia‐related physical decline remain incompletely understood, particularly with respect to the role of motoneuron function. The conceptual definition of sarcopenia has evolved from a narrow focus on muscle mass loss to a broader recognition of its multidimensional nature, with most definitions including reductions in muscle strength and impairments in physical function [1, 2, 3, 4]. Notably, the association between muscle mass and function in older adults [5] is often weak or absent [5], suggesting that additional neuromuscular mechanisms contribute meaningfully to sarcopenia‐related disability. Emerging evidence implicates central and spinal motor pathway dysfunction in the age‐related decline of muscle strength and physical function [6, 7]. Force generation at the motor unit level depends not only on peripheral muscle properties but also on motoneuron discharge rates [8], which are governed by the balance between synaptic input and intrinsic motoneuron excitability [9]. With ageing, motoneurons exhibit reduced discharge rates (particularly at higher contraction intensities [10]), likely reflecting selective degeneration of higher‐threshold motor units [11]. In support of this, there is evidence that older adults with impaired strength production exhibit significantly slower quadriceps motoneuron discharge rates, which explain up to 25% of the variance in their leg muscle strength [12]. These findings shift the focus from peripheral to neural contributors and underscore intrinsic motoneuron excitability as a candidate mechanism linking sarcopenia and functional decline.

A key intrinsic mechanism regulating motoneuron output is the generation of persistent inward currents (PICs), which are modulated by serotonergic and noradrenergic drive onto spinal motoneuron dendrites [13]. PICs amplify (up to 5‐fold), prolong and accelerate motoneuron discharge in response to a given synaptic input [14], scaling with contraction intensity [15, 16]. Without motor neurone excitability modulation by PICs, muscle force would fall below half of maximal [17]. PICs are attenuated in older adults, particularly in those who are physically inactive [18]. Moreover, soleus motoneurons in physically inactive older adults fail to upregulate PICs when transitioning from low to moderate contraction intensities [19], potentially impairing their capacity to perform everyday tasks requiring moderate‐to‐high force generation. Several studies highlight the clinical relevance of intrinsic motoneuron excitability [19, 20, 21]. Pharmacological enhancement of PICs via 5‐HT_2c_ receptor agonism improves muscle strength and physical function in aged mice [20]. In humans, changes in delta frequency (Δ*F*) [14](a noninvasive index of PIC amplitude)correlate with strength recovery following short‐term unloading in young adults [21] and after resistance training in older adults [19]. These findings suggest that intrinsic excitability declines with age, could be further impaired in individuals with reduced physical function and is responsive to interventions. However, it remains unknown whether individuals with sarcopenia demonstrate a distinct excitability profile compared to nonsarcopenic peers and high‐functioning older athletes.

The primary aim of this study was to determine whether intrinsic motoneuron excitability, measured via Δ*F*, differs across the following three distinct ageing phenotypes: older adults with sarcopenia, nonsarcopenic controls and masters athletes. We further examined whether Δ*F* varied across contraction intensities and motor unit recruitment thresholds and whether participants could modulate Δ*F* with increasing contraction demands. We hypothesized that sarcopenic individuals would show reduced Δ*F* and brace height values, particularly at higher contraction intensities and recruitment thresholds, whereas masters athletes would display enhanced excitability and modulation capacity. A secondary exploratory aim was to evaluate the relationships between Δ*F*, muscle strength, power and physical function across this heterogeneous cohort. In addition, we explored potential sex differences and compared subgroups of endurance‐ and power‐trained masters athletes.

## 2 Methods

### 2.1 Participants

This cross‐sectional study included the following three groups of older adults: sarcopenic, nonsarcopenic controls and master athletes. Eligibility criteria for all participants included: age ≥ 65 years; no use of medications known to directly affect the noradrenergic and serotonergic systems (i.e., psychiatric medication); no current use of sex hormone replacement therapy or performance‐enhancing drugs; and no lower body musculoskeletal or neurological conditions (other than sarcopenia) that could impact testing outcomes.

To qualify as sarcopenic, individuals had to meet diagnostic criteria established by the Sarcopenia Definitions and Outcomes Consortium (SDOC) [4] and have a body mass index (BMI) ≤ 30 kg/m^2^. Masters athletes were required to have a minimum of 10 years of competitive sport participation (allowing for < 1 year of interruption) and to have competed within the past year. Athletes were classified as endurance‐type (e.g., long distance running or cycling) or power‐type (e.g., sprinting or weightlifting) sport modalities for subgroup exploratory analysis (Supplementary Material S3). Controls had to be nonsarcopenic and report engaging in no more than one structured exercise session per week in the past year.

Participants were recruited via social media advertisements, a research participant database (with prior consent for recontact), local sporting clubs across the greater Melbourne region (Australia) and retirement communities near Deakin University (Burwood). The study was approved by the Deakin University Human Research Ethics Committee (2023‐241) and conducted in accordance with the Declaration of Helsinki. All participants provided written informed consent prior to enrolment.

### 2.2 Sarcopenia Screening

Participants were screened for sarcopenia using a multistep process. All participants initially completed the five‐item SARC‐F questionnaire [22]. Those with scores ≥ 2 were contacted for follow‐up screening, which included additional questions regarding walking ability and load carrying, as well as an online (video‐based) administration of the Five Times Sit‐to‐Stand (5STS) test. Individuals who reported slowness, difficulty carrying small loads and demonstrated a 5STS time >15 s [1] were invited for in‐person screening of sarcopenia.

A diagnosis of sarcopenia was based on an assessment of muscle strength and physical function according to the SDOC criteria [4]. Muscle strength was assessed using a handheld dynamometer (Jamar Plus, Digital Hand Dynamometer). Participants were seated with the shoulder in anatomical position and elbow flexed to 90° and were instructed to maximally squeeze the dynamometer for 3–5 s using their dominant hand [23]. Two trials were performed, and the highest value was recorded. Physical function was assessed via usual gait speed (UGS) measured over a 4‐m segment using electronic timing gates (Swift Speedlink Performance Equipment, Australia). Participants walked a 6‐m course at their usual pace; UGS was calculated using the time recorded between metres 1 and 5. Participants with handgrip strength <20 kg (females) or <35.5 kg (males), combined with UGS < 0.8 m·s^−1^, were categorized as sarcopenic [4].

### 2.3 Muscle Strength, Power and Physical Function

All eligible participants completed the following tests on‐site: i) Fast gait speed (FGS), measured identically to UGS but performed at maximal walking speed without running; ii) Timed Up and Go (TUG), in which participants stood from a 45 cm chair (without using their hands), walked around a cone positioned 3 m away, returned and sat down as quickly as possible without running; iii) Four‐Square Step Test (4SST), which involved stepping in a predefined sequence (forward, sideways, backward, sideways) through four quadrants outlined by perpendicular canes, completing one clockwise and one counter clockwise trial and iv) 5STS, in which participants stood up fully and sat down five times as quickly as possible from a 45‐cm chair [24]. 5STS time, combined with body mass, height and seat height, was used to estimate lower limb power [25]. Handgrip strength was also measured in all participants as described above. Each test was preceded by standardized verbal instructions, one or more practice trials and a single recorded trial.

Self‐reported function and disability were assessed via the following: i) Katz Activities of Daily Living Scale, evaluating independence in basic tasks such as bathing, dressing and feeding^S1^ and ii) Late‐Life Function and Disability Instrument (LLFDI), which includes a disability component (limitations in life roles/tasks) and a function component (capability for physical tasks)^S2^.

### 2.4 Body Composition

Body composition was assessed using bioelectrical impedance spectrometry (ImpediMed, SOZO, Australia)^S3^. Outcomes included: total body fat mass (kg and %), total body and appendicular skeletal muscle mass (kg) and phase angle (degrees).

### 2.5 Physical Activity

Physical activity was assessed using the International Physical Activity Questionnaire for Elderly (IPAQ‐E), a validated self‐report instrument capturing sitting time, walking and moderate‐to‐vigorous physical activity (all min/day) among older adults^S4^.

### 2.6 Neuromuscular Assessments

Neuromuscular assessments commenced approximately 10 min after completion of the strength, power, functional and body composition testing. Participants were seated upright with their preferred leg secured to an ankle dynamometer (full scale, 226.8 kg; accuracy ±1% full scale; sensitivity 20 mV/V; DinamometroGC, OT Bioelettronica, Italy) positioned at ~70° hip flexion, 0° knee extension and 5° ankle plantarflexion. The foot was strapped just below the proximal phalanx, and the knee was secured to prevent flexion. Following familiarization with dorsiflexion ramped contraction tasks and real‐time feedback, skin preparation was performed, and participants rested for 10–15 min before data collection. The skin over the tibialis anterior was shaved, abraded with degreaser preparation paste (Everi, Spes Medica, Italy), and cleansed with 70% isopropyl alcohol. Two 64‐channel electrode grids (8‐mm interelectrode distance; HD08MM1106, OT Bioelettronica, Italy) were placed over the superior and inferior tibialis anterior using bi‐adhesive foam layers and conductive paste (AC cream, Spes Medica, Italy). A high‐stretch compression bandage (Elastoplast, Australia) ensured stable electrode contact. A dampened strap electrode (WS1.1, OT Bioelettronica) was placed around the ankle joint as a reference electrode. The tibialis anterior was selected for this study due to its high suitability for surface HD‐EMG decomposition and its relevance to age‐related motor decline. The tibialis anterior consistently yields a greater number of decomposable motor units than other lower limb muscles (e.g., soleus, quadriceps) [18, 26, 27], which was particularly important given the expected reduction in motor unit yield in the sarcopenic group. Tibialis anterior is highly relevant for balance/postural control^S5^ and dorsiflexion (foot clearance) during gait^S6^, as well as its activation/strength are associated with the ability to restore balance^S7,S8,S9^ and contribute to the ability to rise from a chair (sit‐to‐stand performance)^S10^; Thus, it contributes to functional capacity, balance and fall prevention in older adults. Prior research also shows that tibialis anterior discharge rates and Δ*F* are markedly reduced with ageing, making it a sensitive muscle for detecting neuromuscular changes in older adults [10, 26].

**FIGURE 1:**
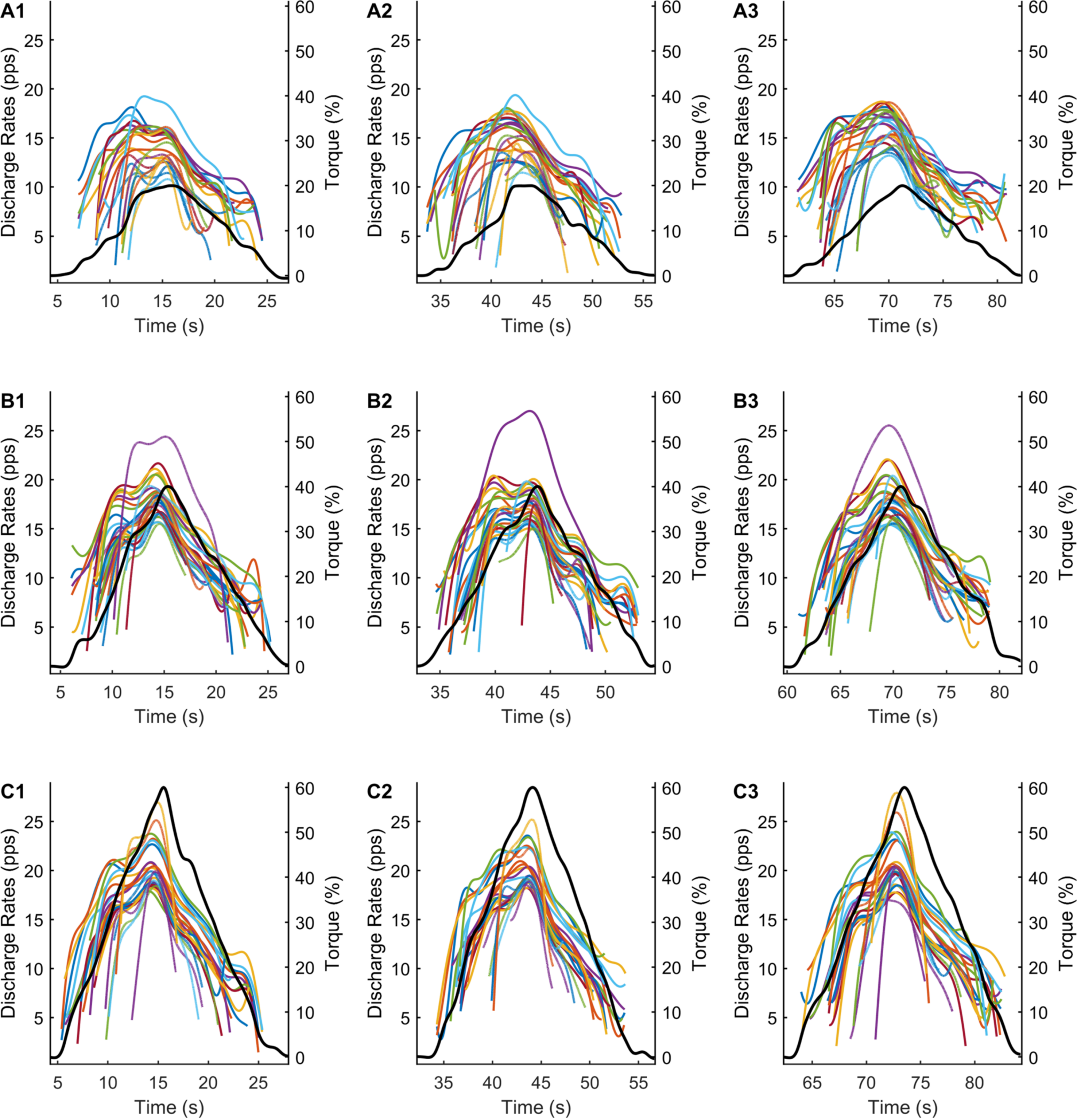
**Experimental design—single participant data (male, control group).** Participants performed three ramp‐shaped contractions to 20% (Panel A, 1–3), 40% (Panel B, 1–3) and 60% (Panel C, 1–3) of their peak torque. Torque is displayed as a continuous black trace. Coloured traces represent the smoothed discharge rates of the motor units decomposed during the respective contractions.

Participants performed three maximal voluntary contractions, with the peak force used to define submaximal targets. They then completed triangular‐shaped ramped contractions at 20%, 40% and 60% (i20%, i40% and i60%) of their maximal voluntary torque (MVT), with force increasing and decreasing at rates of 2%, 4% and 6% of MVT per second, respectively (10s up, 10s down). Up to three trials were performed per intensity, with up to two additional attempts allowed for i20% and i40% if necessary (Figure 1). Rest periods of 90, 120 and 150 s were provided between contractions at 20%, 40% and 60% intensities, respectively. The i20% and i40% contractions were administered in randomized order, followed by the i60% contraction. During these submaximal contractions, surface electromyograms were recorded in monopolar mode, amplified (205×), band‐pass filtered (10–500 Hz), and digitized at 2000 Hz via a 16‐bit wireless amplifier (Muovi+, OT Bioelettronica, Italy) and OTBioLab+ software (v1.5.9.2, OT Bioelettronica, Italy), which also provided real‐time visual force feedback.

### 2.7 Torque and HD‐EMG Analyses

The load cell signal from the ankle dynamometer was digitized, amplified (205×), and low‐pass filtered at 15 Hz prior to conversion into torque values, following manufacturer instructions.

HD‐EMG data were processed offline using DEMUSE software [28]. Signals were band‐pass filtered (20–500 Hz) using a second‐order, zero‐lag Butterworth filter. Decomposition was performed using the convolutive kernel compensation algorithm [28], which yielded motor unit spike trains from each triangular contraction. Identified motor unit filters were then applied to concatenated HD‐EMG signals to track motor units across repeated trials of the same intensity [29]. Motor units were tracked within, but not across, contraction intensities to maximize unit identification and include higher‐threshold units at the higher intensity contractions. After tracking and removing duplicates, a trained investigator (LBRO) manually reviewed and edited the spike trains, and only units with a pulse‐to‐noise ratio ≥30 dB were included.

**FIGURE 2:**
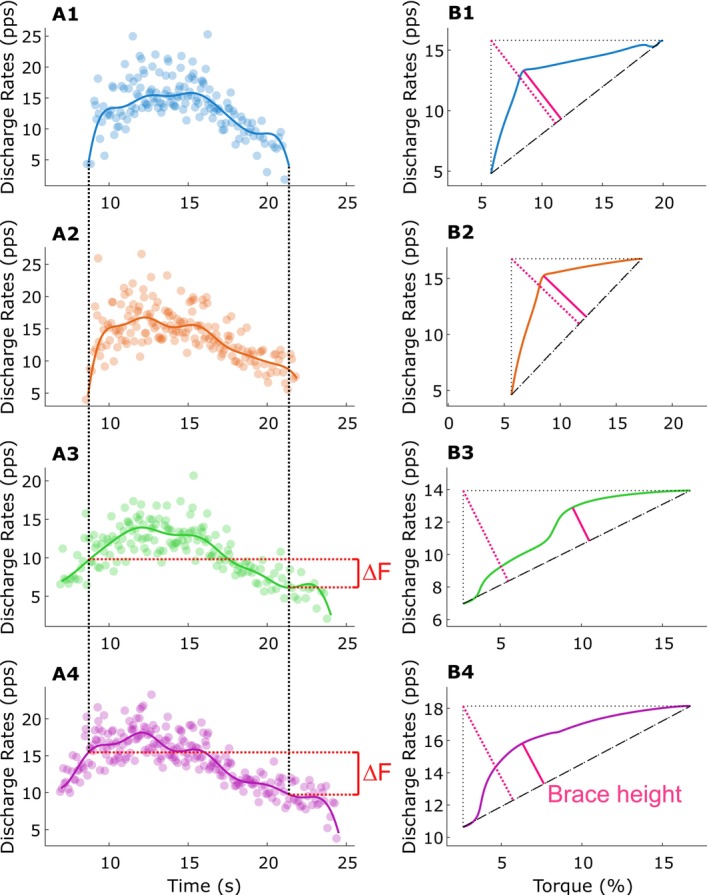
**Paired motor unit analysis for Δ frequency (Δ*F*) calculation and quasi‐geometric analysis for brace height estimation.** The left section (A1–A4) displays smoothed discharge rates (solid lines) and raw instantaneous discharge rates (dots) from four representative motor units recorded during a submaximal isometric contraction up to 20% of the participant's peak torque. A ‘test’ motor unit is shown in Panel A1, and potential ‘control’ units in Panels A3 and A4. Panel A2 shows a motor unit that did not meet the criteria to serve as a control unit. Vertical dashed lines indicate the recruitment and derecruitment times of the test unit, which define the window for Δ*F* calculation. In Panels A3 and A4, the smoothed discharge rates of the control units intersect with the test unit's recruitment and derecruitment times. The difference in discharge rate of the lower‐threshold control unit at the time of recruitment and derecruitment of the higher‐threshold test unit (i.e., recruitment–derecruitment hysteresis) defines Δ*F*, shown as red brackets. Each test unit was paired with all eligible control units, and the corresponding Δ*F* values were averaged to yield a single Δ*F* value per test unit per contraction. The right section (B1–B4) shows the ascending phase of the smoothed discharge rate plotted as a function of torque (% of peak) for the same motor units shown in section A. A linear discharge trace (black dashed line) is fitted from recruitment to peak discharge. Brace height (magenta line) is defined as the maximal perpendicular distance between the observed smoothed discharge rate trajectory and the linear discharge trace. This metric quantifies the deviation from a linear acceleration pattern and theoretically reflects neuromodulatory input onto motor neurons. Pink dashed lines represent the theoretical maximum brace height vector used for normalization.

Instantaneous discharge rates were calculated from spike times, and continuous (smoothed) discharge rate curves were estimated using support vector regression (Figure 1) [30]. Paired motor unit analysis was used to estimate PICs, in which a lower‐threshold ‘control’ unit was paired with a higher‐threshold ‘test’ unit according to previously defined criteria [31]. Delta frequency (Δ*F*) was defined as the difference in discharge rate of the control unit at test unit recruitment versus derecruitment, reflecting recruitment–derecruitment hysteresis and indexing PIC amplitude [14] (Figure 2).

Brace height, a marker of motor unit discharge rate neuromodulation, measures the discharge rate deviation of linearity during the ascending phase of the ramped contraction. Brace height was calculated as the maximum orthogonal deviation between actual discharge rate and a linear fit from motor unit recruitment to peak discharge. This value was normalized to the hypotenuse of a right triangle formed between these two points [32] (Figure 2). Motor units with negative recruitment‐to‐brace height slopes or normalized brace heights exceeding 100% were reviewed and excluded if deemed erroneous [32].

Peak discharge rate was defined as the maximum value of the smoothed discharge rate, and recruitment threshold was defined as the torque (%MVT) at which the first discharge occurred. For statistical analyses, motor units were categorized into the following three recruitment threshold bins: i) rt0%–20%: recruitment threshold ≤20% MVT; ii) rt20%–40%: recruitment threshold >20 and ≤40% and iii) rt40%–60%: recruitment threshold >40 and ≤60%.

### 2.8 Statistical Analysis

Robust statistical models were used to address potential violations of classical assumptions, such as the presence of outliers and heteroscedasticity. These models rely on M‐estimation, which minimizes the influence of extreme observations, prioritizing accurate parameter estimation over null hypothesis significance testing. Interpretation is based on regression slopes and 95% confidence intervals (CIs), as *p* values depend on distributional assumptions that are often violated in robust analyses.

Group differences in continuous variables were analyzed using robust linear models, accounting for sex. For IPAQ‐E outcomes, where a substantial proportion of participants reported no moderate‐to‐vigorous activity, analyses followed a two‐step procedure. First, robust logistic regression was used to model the likelihood of engaging in any moderate or vigorous physical activity (zero vs. nonzero). Second, among those reporting nonzero activity, robust gamma regression with a log link was used to compare duration (minutes/day) of sitting, walking and light‐to‐vigorous activity across groups.

Separate robust linear mixed‐effects models for each motor unit recruitment threshold bin (rt0%–20, rt20%–40% and rt40%–60%) tested group differences in HD‐EMG outcomes across contraction intensities (i20%, i40%, i60%) and their interaction, adjusting for sex (fixed effect). Motor units were nested within participants, and random intercepts were included to account for intra‐individual correlation. Post hoc group comparisons were performed using nonparametric bootstrapping with 2000 resamples, applying the bias‐corrected and accelerated method to generate 95% CIs. This method adjusts for both bias and skewness in the bootstrap distribution, yielding more accurate estimates. The impact of including age as a covariate in the ∆F models was also examined, with results presented in Supplementary Material S2.

Robust linear mixed‐effects models were used to assess the relationship between Δ*F* and muscle strength, power and physical function measures, including interaction terms for biological sex and contraction intensity. Motor units were again nested within participants, with a random intercept to account for repeated measures. Model coefficients (β) and 95% CIs were estimated, adjusting for body mass when appropriate. Predictive *R* ^2^ values were calculated as the squared correlation between observed and predicted outcomes, reported both marginally (*R* ^2^m; variance explained by fixed effects alone) and conditionally (*R* ^2^c; variance explained by both fixed and random effects). The *R* ^2^m reflects the proportion of the outcome variability that is captured by the predictors of primary interest, whereas the *R* ^2^c reflects how much variability is captured when both these predictors and individual‐specific effects are taken into account.

All analyses and figure generation were conducted using RStudio (version 2024.04.2) and MATLAB (version 2023b, The MathWorks, Natick, MA, USA). The dataset, R code and packages information can be found at: https://github.com/orssatto/PICs‐sarcopenia.

## 3 Results

### 3.1 Participant Characteristics

Fifty‐six participants were enrolled (50% females), including 12 with sarcopenia, 23 nonsarcopenic controls and 21 masters athletes (Table 1). On average, sarcopenic participants were approximately 5 years older than controls and 10 years older than masters athletes. Although age did not differ significantly between sarcopenic participants and controls or between controls and masters athletes, sarcopenic participants were significantly older than masters athletes. BMI was significantly lower in the masters athletes compared to both the sarcopenic (−15%) and the control groups (−12%) (Table 1).

**TABLE 1:** Participant characteristics.

Characteristics	Sarcopenic	Controls	Athletes
Females, *n*	7 [58.3]	13 [56.5]	8 [38.1]
Age, years	79.9 (76.2, 83.7)	74.5 (71.8, 77.2)	69.8 (66.9, 72.6)
Height, m	1.64 (1.59, 1.68)	1.67 (1.64, 1.70)	1.69 (1.66, 1.72)
Body mass index, kg/m^2^	26.4 (24.3, 28.5)	25.7 (24.2, 27.2)	22.5 (20.9, 24.1)
Underweight, *n*	2 [16.7]	0 [0.0]	0 [0]
Normal weight, *n*	3 [25.0]	8 [34.8]	16 [76.2]
Overweight, *n*	7 [58.3]	12 [52.2]	5 [23.8]
Obese, *n*	0 [0.0]	3 [13.0]	0 [0]
**Medication use**			
Medication use, *n*	1 [1, 4]	1 [1, 3]	0 [0, 1]
**Physical activity**			
Sitting, min/day	360 [180, 480]	360 [300, 390]	360 [360, 480]
Walking, min/day	22 [11. 43]	40 [26, 60]	60 [40120]
Moderate activity, min/day	3.1 [0, 9.3]	22.9 [8.6, 55.7]	25.7 [12.9, 120]
Vigorous activity, min/day	0 [0, 0]	0 [0, 9]	26 [17, 103]

Values are estimated marginal means (95% CI lower and upper limits), number (*n*) with proportion [%] or median [1st and 3rd quartiles].

The masters athletes included 10 individuals in predominantly power‐based sports (e.g., power/weightlifting, sprinting) and 11 endurance‐based sports (e.g., distance running, race walking, cycling). On average, the athletes reported 45.8 years (range 20–72) of sports participation, a median of four competitions in the past year (range 1–12), and trained an average of five sessions per week (range 3–10) in the past year, with an average session duration of 63 min (range 40–120). Fifteen (71%) reported incorporating some form of resistance training into their training routines.

There were no significant differences in sitting time, but Masters athletes reported significantly higher levels of walking, moderate and vigorous physical activity than both other groups. No significant differences were observed between the sarcopenia and control groups in these measures (Table 1).

#### 3.1.1 Physical Function

The sarcopenic group demonstrated significantly poorer performance on FGS, 4SST, TUG, 5STS and peak torque than both controls (mean difference, −30% to −56%) and athletes (−42% to −55%) (Table 2). Masters athletes exhibited on average an 11%–23% better performance on FGS, 4SST and TUG and higher 5STS‐derived power than controls (Table 2).

**TABLE 2:** Participants physical function and body composition per group and estimated marginal mean differences between groups.

	Estimated marginal means (95% CI)			Estimated marginal mean differences (95% CI)		
Physical function	Sarcopenic	Controls	Athletes	Sarcopenic—controls	Sarcopenic—athletes	Athletes—controls
Handgrip strength, kg	22.9 (19.7, 26.1)	32.5 (30.2, 34.8)	34.6 (32.1, 37.1)	**−9.6** **(−14.4, −4.9)**	**−11.7** **(−16.6, −6.8)**	2.1 (−2.0, 6.2)
UGS, m·s^−1^	0.77 (0.68, 0.86)	1.38 (1.32, 1.45)	1.38 (1.31, 1.45)	**−0.61** **(−0.74, −0.48)**	**−0.61** **(−0.74, −0.47)**	0.00 (−0.12, 0.11)
FGS, m·s^−1^	1.04 (0.90, 1.20)	1.93 (1.82, 2.03)	2.15 (2.04, 2.27)	**−0.89** **(−1.10, −0.67)**	**−1.11** **(−1.34, −0.89)**	**0.23** **(0.04, 0.41)**
4SST, s	12.55 (11.78, 13.33)	8.78 (8.24, 9.32)	7.26 (6.69, 7.83)	**3.77** **(2.65, 4.90)**	**5.29** **(4.14, 6.45)**	**−1.52** **(−2.46, −0.58)**
TUG, s	12.23 (11.73, 12.73)	6.72 (6.36, 7.08)	5.81 (5.43, 6.19)	**5.51** **(4.78, 6.25)**	**6.42** **(5.67, 7.17)**	**−0.91** **(−1.54, −0.28)**
5STS, s	17.0 (16.1, 17.7)	11.1 (10.5, 11.7)	9.4 (8.8, 10.1)	**5.8** **(5.2, 6.5)**	**7.5** **(6.8, 8.2)**	**−1.6** **(−2.2, −1.1)**
5STS, W·kg^−1^	1.91 (1.63, 2.20)	3.02 (2.82, 3.23)	3.72 (3.51, 3.94)	**−1.11** **(−1.52, −0.69)**	**−1.81** **(−2.24, −1.38)**	**0.70** **(0.34, 1.06)**
Dorsiflexion peak torque, N·m	11.4 (7.9, 14.8)	26.2 (23.7, 28.7)	25.6 (23.0, 28.3)	**−14.9** **(−20.0, −9.7)**	**−14.3** **(−19.5, −9.0)**	−0.6 (−5.0, 3.8)
Dorsiflexion peak torque/body mass, N·m·kg^−1^	0.164 (0.112, 0.216)	0.372 (0.334,0.410)	0.392 (0.352,0.432)	**−0.208** **(−0.285, −0131)**	**−0.228** **(−0.307, −0.149)**	0.020 (−0.046, 0.086)
**Body composition**						
Body mass, kg	69.4 (62.8, 76.0)	71.6 (66.8, 76.4)	64.4 (59.4, 69.4)	−2.2 (−11.9, 7.5)	5.0 (−5.0, 15.0)	−7.2 (−15.5, 1.2)
Total body fat mass, kg	22.8 (18.9, 26.7)	20.9 (18.0. 23.8)	12.8 (9.8, 15.8)	1.9 (−3.9, 7.7)	**10.1** **(4.1, 16.0)**	**8.2** **(3.1, 13.2)**
Body fat %	32.3 (27.9, 36.6)	29.0 (25.8, 32.2)	20.0 (16.7, 23.3)	3.3 (−3.2, 9.7)	**12.3** **(5.7, 18.9)**	**−9.0** **(−14.6, −3.4)**
Total body SMM, kg	20.3 (19.0, 21.7)	22.1 (21.0, 23.1)	23.3 (22.2, 24.3)	−1.7 (−3.7, 0.3)	**−2.9** **(−5.0, −0.8)**	1.2 (−0.5, 3.0)
ASMM, kg	13.6 (12.7, 14.5)	15.0 (14.4, 15.7)	14.5 (13.8, 15.2)	**−1.4** **(−2.8, −0.1)**	−0.9 (−2.2, 0.5)	−0.6 (−1.7, 0.6)
Phase angle, degrees	3.76 (3.45, 4.08)	4.69 (4.46, 4.92)	5.30 (5.06, 5.54)	**−0.93** **(−1.39, −0.46)**	**−1.54** **(−2.01, −1.06)**	**0.61** **(0.21, 1.02)**
**LLFDI**						
Disability component—Frequency dimension, %	54.2 (57.0, 61.7)	54.6 (52.4, 56.8)	59.4 (57.0, 61.7)	−0.4 (−5.0, 4.0)	−5.2 (−9.9, 0.5)	**4.8** **(0.8, 8.7)**
Disability component – Limitation dimension, %	63.9 (55.3, 72.5)	80.7 (74.5, 87.0)	90.4 (83.9. 97.0)	**−16.8** **(−29.5, −4.1)**	**−26.5** **(−39.6, −13.5)**	9.7 (−1.2, 20.6)
Function component—total score, %	56.4 (49.2, 63.6)	73.4 (68.5. 78.4)	87.8 (82.6, 93.0)	**−17.0** **(−27.4, −6.6)**	**−31.4** **(−42.2, −20.7)**	**14.4** **(5.7, 23.1)**

*Note:* Bolded estimated marginal mean differences (95% CI) highlight confidence intervals not crossing zero, indicating statistical differences.

Abbreviations: 4SST, 4‐Square Step Test; 5STS, 5‐time Sit‐to‐Stand; ASMM, appendicular skeletal muscle mass; FGS, fast gait speed; LLFDI, Late‐life Functional Disability Instrument; SMM, skeletal muscle mass; TUG, Timed Up and Go; UGS, usual gait speed.

#### 3.1.2 Anthropometry and Body Composition

Height and body mass did not differ significantly across groups, although body mass was on average 7.2 and 5.0 kg higher in the control and sarcopenia groups, respectively, compared to masters athletes (Tables 1 and 2). Athletes had lower body fat percentages. Total SMM was 14.8% greater in athletes compared to individuals with sarcopenia. ASMM was 10% lower in those with sarcopenia compared to controls. Phase angle was highest in athletes and lowest in those with sarcopenia (Table 2).

#### 3.1.3 Self‐Reported Function

For the LLFDI disability component (frequency domain), both sarcopenia and control groups scored lower than the athlete group (Table 2). For the limitations domain, the sarcopenia group scored lower than both the control and athletes. For the function component, the sarcopenia group again scored lowest, followed by controls, with athletes scoring the highest (Table 2). For the Katz Index of Activities of Daily Living survey, all participants reported a score of zero (indicating full independence), except three individuals in the sarcopenia group, who reached a score of 1.

### 3.2 Motor Unit Identification

A total of 13 685 spike trains from 4998 unique motor units were successfully decomposed and included in the analyses. Supplementary material S1 describes the distribution of motor units by sex, group, contraction intensity and recruitment threshold.

### 3.3 Intrinsic Motoneuron Excitability (∆*F*) Differentiates Sarcopenic, Nonsarcopenic and Athletic Older Adults, Particularly at Higher Thresholds and Intensities

**FIGURE 3:**
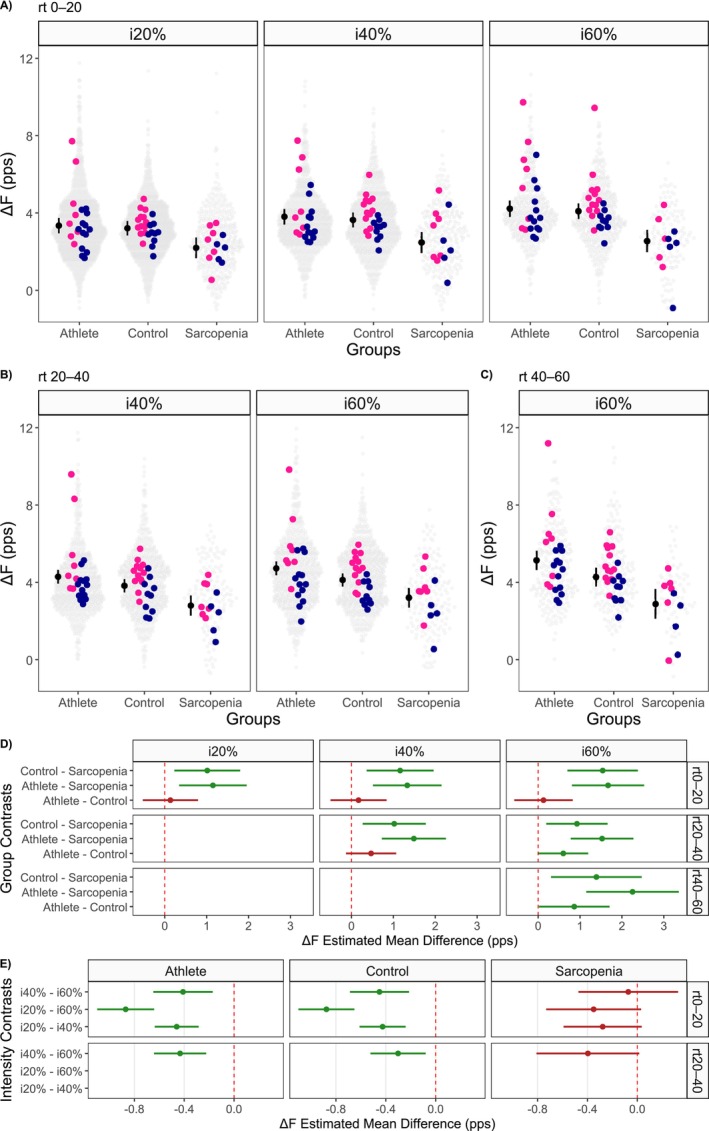
**Intrinsic motor neurone excitability (∆*F*).** Panels A, B and C present data stratified by recruitment threshold bins: 0%–20%, 20%–40% and 40%–60%, respectively. Each panel displays Δ*F* values across groups (master athletes, controls and sarcopenia) and contraction intensities (20%, 40% and 60% of participants' maximal force). Black circles and lines represent estimated marginal means with their corresponding 95% confidence intervals. Average Δ*F* values for each female and male participant are shown as pink and blue circles, respectively. Transparent grey dots indicate individual motor unit data points. Panel D shows group contrasts in Δ*F* at each contraction intensity, while Panel E illustrates Δ*F* contrasts across contraction intensities within each group. Circles and lines in Panels D and E represent estimated marginal mean differences and their 95% confidence intervals. Green indicates confidence intervals that do not cross zero (statistical significance), while red indicates nonsignificant findings.

In low‐threshold units (rt0%–20%), a significant group‐by‐intensity interaction was observed (β = −0.52 [−0.89, −0.15], *t* = −2.74). Sarcopenic individuals had significantly lower Δ*F* values compared to controls (−32% to −38%) and athletes (−34% to −40%) at i20%, i40% and i60%. No significant differences were observed between athletes and controls. Δ*F* increased with contraction intensity in athletes and controls but remained unchanged in sarcopenia (Figure 3, Table 3).

**TABLE 3:** Delta frequency **(∆** *F*), brace height and peak discharge rates estimated marginal mean and mean differences (95% confidence intervals) across groups, contraction intensities and motor unit recruitment thresholds.

	Estimated marginal means (95% CI)			Estimated marginal mean differences (95% CI)		
	Sarcopenic	Controls	Athletes	Sarcopenic—controls	Sarcopenic—athletes	Athletes—controls
**∆*F* (pps)**						
**Contraction intensity i20%**						
Recruitment threshold 0%–20%	2.20 (1.66, 2.74)	3.22 (2.84, 3.59)	3.35 (2.95, 3.75)	**−1.01** **(−1.80, −0.23)**	**−1.15** **(−1.96, −0.34)**	0.14 (−0.52, 0.79)
**Contraction intensity i40%**						
Recruitment threshold 0%–20%	2.48 (1.93, 3.03)	3.64 (3.28, 4.03)	3.81 (3.41, 4.21)	**−1.16** **(−1.96, −0.36)**	**−1.33** **(−2.15, −0.51)**	0.17 (−0.49, 0.84)
Recruitment threshold 20%–40%	2.81 (2.28, 3.34)	3.83 (3.48, 4.18)	4.30 (3.94, 4.66)	**−1.02** **(−1.77, −0.27)**	**−1.49** **(−2.25, −0.73)**	0.47 (−0.13, 1.07)
**Contraction intensity i60%**						
Recruitment threshold 0%–20%	2.55 (1.97, 3.13)	4.09 (3.69, 4.49)	4.22 (3.80, 4.64)	**−1.54** **(−2.38, −0.70)**	**−1.67** **(−2.53, −0.81)**	0.13 (−0.57, 0.83)
Recruitment threshold 20%–40%	3.21 (2.70, 3.72)	4.13 (3.79, 4.47)	4.73 (4.37, 5.09)	**−0.93** **(−1.66, −0.19)**	**−1.53** **(−2.27, −0.78)**	**0.60** **(0.01, 1.19)**
Recruitment threshold 40%–60%	2.89 (2.12, 3.66)	4.28 (3.79, 4.77)	5.14 (4.64, 5.64)	**−1.39** **(−2.47, −0.31)**	**−2.25** **(−3.36, −1.15)**	**0.86** **(0.02, 1.71)**
**Brace height (%rTri)**						
**Contraction intensity i20%**						
Recruitment threshold 0%–20%	35.7 (32.4, 38.9)	33.3 (35.7, 38.9)	33.0 (30.6, 35.4)	2.3 (−2.4, 7.1)	2.7 (−2.2, 7.5)	−0.4 (−4.3, 3.6)
**Contraction intensity i40%**						
Recruitment threshold 0%–20%	35.3 (32.0, 38.6)	31.3 (29.1, 33.6)	32.7 (30.3, 35.1)	4.0 (−0.8, 8.7)	2.6 (−2.3, 7.5)	1.4 (−2.6, 5.4)
Recruitment threshold 20%–40%	20.9 (17.2, 24.6)	20.3 (18.2, 22.4)	21.2 (19.1, 23.3)	0.6 (−4.4, 5.7)	−0.3 (−5.3, 4.8)	0.9 (−2.7, 4.5)
**Contraction intensity i60%**						
Recruitment threshold 0%–20%	30.8 (27.3, 34.2)	28.4 (26.0, 30.8)	30.0 (27.5, 32.6)	2.4 (−2.6, 7.4)	0.7 (−4.4. 5.9)	1.7 (−2.5, 5.8)
Recruitment threshold 20%–40%	23.4 (20.3, 26.6)	26.4 (24.7, 28.2)	27.7 (25.8. 29.6)	−3.0 (−7.3, 1.3)	−4.3 (−8.7, 0.1)	1.3 (−1.9, 4.4)
Recruitment threshold 40%–60%	18.1 (13.5, 22.7)	17.6 (14.4, 20.9)	19.9 (17.1, 22.7)	0.5 (−6.1, 7.1)	−1.8 (−8.3, 4.7)	2.3 (−2.9, 7.5)
**Peak discharge rates (pps)**						
**Contraction intensity i20%**						
Recruitment threshold 0%–20%	12.4 (11.1, 13.7)	13.5 (12.6, 14.4)	13.7 (12.7, 14.7)	−1.1 (−3.0, 0.8)	−1.3 (−3.3, 0.6)	0.2 (−1.4, 1.9)
**Contraction intensity i40%**						
Recruitment threshold 0%–20%	14.0 (12.7, 15.3)	16.2 (15.3, 17.2)	16.2 (15.2, 17.2)	**−2.2** **(−4.1, −0.3)**	**−2.2** **(−4.2, −0.2)**	0.0 (−1.7, 1.6)
Recruitment threshold 20%–40%	13.6 (11.7, 15.5)	16.1 (14.8, 17.5)	15.9 (14.5, 17.4)	−2.6 (−5.3, 0.2)	−2.3 (−5.2, 0.5)	−0.2 (−2.6, 2.2)
**Contraction intensity i60%**						
Recruitment threshold 0%–20%	16.3 (15.0, 17.6)	18.4 (17.4, 19.3)	18.6 (17.6, 19.6)	**−2.1** **(−4.0, −0.1)**	**−2.3** **(−4.3, −0.3)**	0.2 (−1.4, 1.9)
Recruitment threshold 20%–40%	16.3 (14.4, 18.2)	18.5 (17.2, 19.9)	19.2 (17.8, 20.7)	2.3 (−5.1, 0.5)	**−3.0** **(−5.8, 0.1)**	0.7 (−1.7, 3.0)
Recruitment threshold 40%–60%	16.0 (13.5, 18.4)	17.8 (16.2, 19.5)	19.8 (18.1, 21.5)	−1.9 (−5.4, 1.6)	**−3.8** **(−7.4, −0.2)**	2.0 (−0.8, 4.8)

*Note:* Bolded estimated mean differences (95% CI) highlight confidence intervals not crossing zero, indicating statistical differences.

Abbreviations: %rTri, % of right triangle; Pps, pulses per second.

In mid‐threshold units (rt20%–40%), main effects of group (β = −1.49 [−2.13, −0.85], *t* = −4.57) and intensity (β = 0.43 [0.22, 0.64], *t* = 4.06) were observed without interaction. Δ*F* was consistently lower in sarcopenic individuals compared to controls (−22 to −27%) and athletes (−32 to −37%) across all intensities, and Δ*F* increased from i40% to i60% regardless of group (Figure 3, Table 3).

In high‐threshold units (rt40%–60%), a significant group effect was observed at i60% (β = −2.25 [−3.18, −1.33], *t* = −4.78). Sarcopenia showed lower Δ*F* than controls (−32%) and athletes (−44%); athletes also had higher Δ*F* than controls (17%) (Figure 3, Table 3).

Sex was a significant predictor of Δ*F* across all bins. Male participants exhibited lower Δ*F* than females in motor units recruited from 0% to 20% (β = −0.73 [−1.21, −0.24], *t* = −2.95), 20%–40% (β = −1.21 [−1.64, −0.78], *t* = −5.55) and 40%–60% (β = −1.25 [−1.90, −0.61], *t* = −3.79).

The exploratory analyses including age as a covariate revealed it had no significant effect on the model and no meaningful impact on the main effects or interactions. Estimated marginal means remained consistent with the original model; however, CIs were wider, introducing uncertainty in some contrasts. Full results are presented in Supplementary Material S2.

### 3.4 Neuromodulatory Effects on Discharge Rates Do Not Differ Across Groups

In rt0%–20% units, a group‐by‐intensity interaction was observed (β = −2.03 [−3.89, −0.18], *t* = −2.15), although pairwise contrasts revealed no between‐groups differences at any intensity (Table 3). No group‐by‐intensity interaction or main effects were observed for rt20%–40% and rt40%–60%. Sex differences emerged with males exhibiting lower brace heights in the rt0%–20% (β = −5.64 [−8.50, −2.77], *t* = −3.86) and rt20%–40% units (β = −4.09 [−6.22, −1.96], *t* = −3.76).

### 3.5 Peak Discharge Rates Are Inconsistently Lower in Sarcopenic Individuals

In rt0%–20% units, a group‐by‐intensity interaction effect was found (β = −0.98 [−1.46,−0.49], *t* = −3.96). Sarcopenic individuals had significantly lower peak discharge rates than controls and athletes at i40% and i60%. In rt20%–40% units, a group‐by‐intensity interaction effect was found (β = −0.88 [−1.43,−0.33], *t* = −3.14). Peak discharge was lower in sarcopenia compared to athletes in i60%. In rt40%–60% units, a group main effect was observed (β = −3.84 [−6.85,−0.82], *t* = −2.50). Peak discharge was lower in sarcopenia compared to athletes (Table 3). Sex differences were evident in all bins, with males exhibiting lower values in rt0%–20% units (β = −1.27 [−2.49,−0.06], *t* = −2.06), rt20%–40% units (β = −2.31 [−4.05,−0.57], *t* = −2.60) and rt40%–60% units (β = −2.51 [−4.65,−0.38], *t* = −2.31).

### 3.6 Lower Δ*F* Is Associated to Poorer Strength, Power and Function, Especially at Higher Contraction Intensities

**FIGURE 4:**
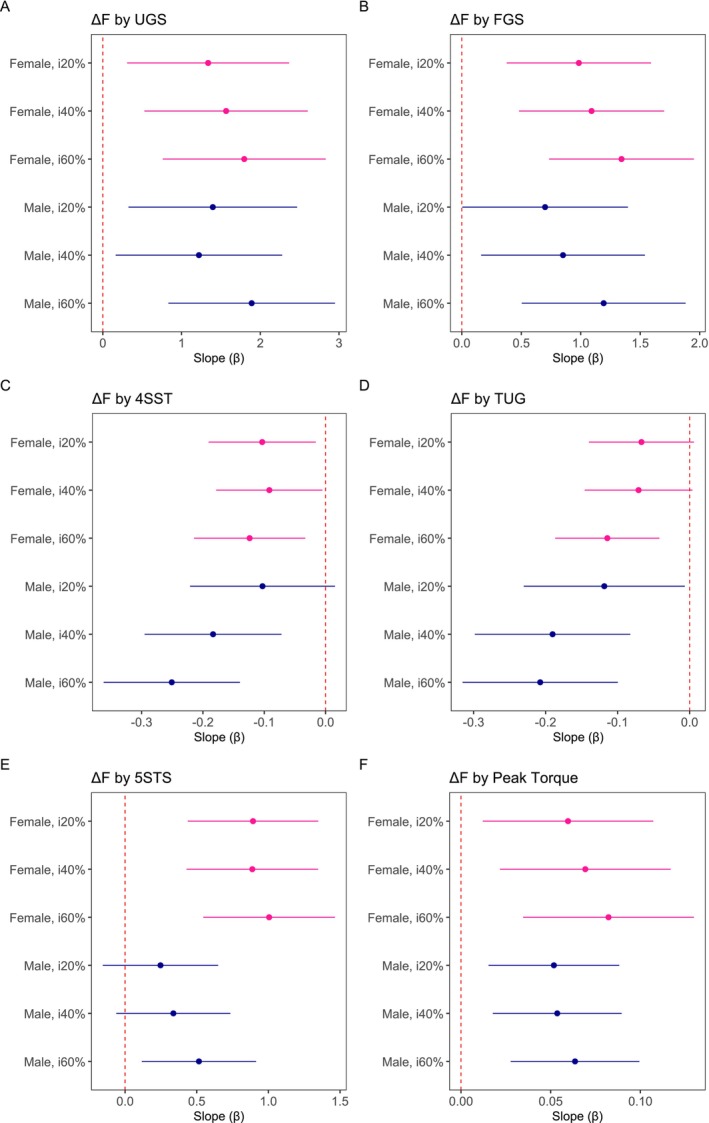
**Slopes of the relationship between Δ*F* and physical function variables by sex and contraction intensity for males and females**. The plot displays slope estimates (β) with 95% confidence intervals for females and males (i20%, i40% and i60% contraction intensities). Significant relationships are observed when the 95% confidence intervals do not cross zero. The slope (β) represents the change in ∆F in relation to change of 1 unit of measure for the respective variables: A) UGS, usual gait speed (m·s^−1^); B) FGS, fast gait speed (m·s^−1^); C) 4SST, 4‐Square Step Test Time (s); D) TUG, Timed Up and Go time (s); E) 5STS, 5‐Times Sit‐to‐Stand estimated power by body mass (W·kg^−1^) and F) peak torque (N·m ^−1^), with models accounting for body mass.

**FIGURE 5:**
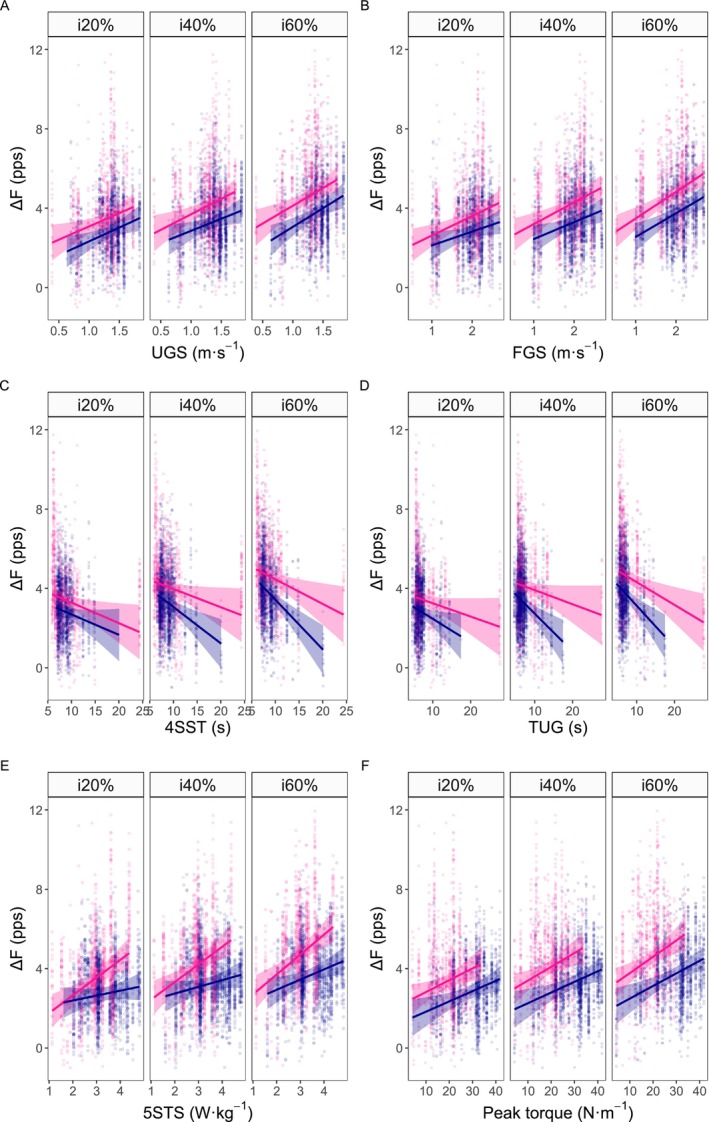
**Association between Δ*F* and measures of physical function, muscle strength and muscle power across sex and contraction intensity.** Panels show robust linear mixed‐effect model predictions of Δ*F* (pps) as a function of: A) usual gait speed (UGS); B) fast gait speed (FGS); C) 4‐Square Step Test (4SST); D) Timed‐Up‐and‐Go (TUG); E) 5‐Repetition Sit‐to‐Stand power (5STS) and F) peak torque, with models accounting for body mass. Regression lines and 95% confidence intervals (shaded areas) are shown separately for females (pink) and males (blue), across contraction intensities (i20%, i40%, i60%). Points represent individual motor unit data. y‐axis range is limited from −1 to 12 for improved slopes visualization.

Figure 4 presents model slopes, representing the change in ∆F in relation to change of 1 unit of measure for strength, power or function. Figure 5 provides scatterplots of ∆F versus strength, power and functional measures. Lower Δ*F* was associated with slower UGS and FGS at all intensities in both sexes (UGS, *R* ^2^c = 0.81 and *R* ^2^m = 0.14; and FGS, *R* ^2^c = 0.81 and *R* ^2^m = 0.16). In females, slower 4SST times were associated with lower Δ*F* across all intensities; in males, the association was strengthened at 40% and 60% (*R* ^2^c = 0.81 and *R* ^2^m = 0.14). Slower TUG time was associated with lower Δ*F* in both sexes but reached significance only at i60% in females; the association was consistent across all intensities in males (*R* ^2^c = 0.81 and *R* ^2^m = 0.14). Reduced 5STS power was associated with lower Δ*F* in females at all intensities, although significance in males was limited to i60% (*R* ^2^c = 0.81 and *R* ^2^m = 0.15). Weaker peak torque normalized to body mass was associated with lower Δ*F* in females across all intensities and in males at i40% and i60% (*R* ^2^c = 0.81 and *R* ^2^m = 0.12).

### 3.7 Exploratory Analysis Comparing Endurance‐ and Power‐Type Masters Athletes

Exploratory analyses comparing endurance and power athlete subgroups (see Supplementary Material S3) suggest that power athletes may exhibit a tendency toward higher Δ*F* values (indicative of greater motoneuron excitability), better performance in some physical function tests, lower body fat percentage and higher phase angle. However, no clear differences were observed in brace height or muscle mass. These findings should be interpreted with caution due to the modest number in each group, which limits statistical power, and primarily serve to inform future study design and sample size estimations.

## 4 Discussion

### 4.1 Main Findings

The key findings from this study were that intrinsic motoneuron excitability is markedly reduced in older adults with sarcopenia, as evidenced by consistently lower Δ*F* values across contraction intensities and recruitment thresholds. Moreover, the sarcopenic group failed to modulate Δ*F* in response to increasing contraction demands, suggesting a blunted or absent PIC response. This excitability deficit was strongly associated with reduced muscle strength and impaired functional capacity, reinforcing the view that sarcopenia is not solely a disorder of the muscle, but one that involves profound neuromuscular dysfunction. Although we expected masters athletes, who exhibited clearly superior functional performance, to show globally elevated Δ*F*, this was only evident at higher intensities and in higher‐threshold motor units. These findings indicate that while intrinsic excitability may remain intact at low activation levels in normal aging, the ability to engage PICs during more demanding contractions may distinguish functional phenotypes.

### 4.2 Intrinsic Motoneuron Excitability

Prior work suggests that Δ*F* is consistently lower in older than in young adults across both upper‐ and lower‐limb muscles [26, 27, 31, 33], collectively establishing that ageing per se is accompanied by a decline in intrinsic motoneuron excitability. Our study extends this work by contrasting distinct ageing phenotypes. Sarcopenic participants exhibited the lowest Δ*F*, implying that the already weakened PICs of normal ageing are especially compromised when clinical sarcopenia is present. This finding aligns with the emerging view that neuromuscular impairments contribute substantially to sarcopenia severity beyond what can be explained by muscle mass loss alone [6, 7]. At the opposite end of the spectrum, masters athletes showed higher excitability at high contraction intensities and recruitment thresholds. The lack of difference between masters athletes and nonathletic controls at i20% aligns with a previous cross‐sectional study in young adults, which found similar Δ*F* values between trained and untrained individuals at that same intensity; however, that study did not assess higher intensities [34]. In contrast, our results partly align with a longitudinal trial in older adults, where 6 weeks of high‐load power training increased soleus Δ*F*, particularly at increasing intensities (i20% vs. i40%) [19]. These observations suggest that lifelong physical training can mitigate age‐related losses in motoneuron excitability, but the benefit becomes more apparent when the neuromuscular system is strongly challenged, that is, when higher intensities and higher‐threshold motor units are recruited. Such differences might have been driven mostly by the power‐type athletes, as exploratory analysis suggests higher excitability compared to endurance athletes (Supplementary Material S3). However, this exploratory analysis must be interpreted with caution due to the small sample size in each group, which likely limited the ability to detect significant and meaningful differences. Another notable pattern in our data is that Δ*F* increases with contraction intensity in both controls and, more markedly, in masters athletes, whereas no such increase was observed in the sarcopenic group. It is expected that, in a healthy system, motoneuron PICs are upregulated as synaptic drive increases, contributing to discharge modulation during strong efforts [15], enabling the system to achieve more than 50% of its maximal force [17]. By contrast, the sarcopenic group's flat Δ*F* across i20–i60% suggests that additional synaptic input (from volitional effort) was likely necessary to achieve the desired force levels in this group. Hence, the capacity to modulate intrinsic excitability appears to be a key discriminator between healthy and pathological ageing.

The pronounced Δ*F* reduction in sarcopenic adults likely reflects combined deficits in cellular, neuromodulatory, inhibitory control and/or structural mechanisms that govern PIC generation. PICs depend primarily on dendritic L‐type Ca^2+^ and Na^+^ channels, which are strongly facilitated by descending serotonergic and noradrenergic input. Ageing compromises both channel function and brain‐stem neuromodulatory pathways. Degeneration of the locus coeruleus and dorsal raphe diminishes noradrenaline and serotonin release, and these deficits may be exacerbated in sarcopenia, where brain‐stem atrophy and chronic systemic inflammation can further blunt neurotransmitter action and monoaminergic receptor's function (see discussion in Orssatto et al. [18]). Age‐related motoneuron degeneration, with preferential damage/loss of large, high‐threshold units [11, 35], could additionally deteriorate channel density and receptor integrity critical for PICs. It is important to note that the brace height results suggest no between‐group differences in neuromodulatory drive to motoneurons. However, it is unclear whether this reflects a lack of sensitivity for group comparisons or whether impaired inhibitory control of PICs underlies our findings. This apparent contradiction can be reconciled by recognizing that while the two measures are related, they are not equivalent. Δ*F* is thought to represent PIC modulation by both neuromodulatory and/or inhibitory inputs, while the brace height seems more sensitive to PIC modulation by neuromodulatory input alone [32]. One of the hypotheses is that the observed reduction in Δ*F* in sarcopenia may not necessarily indicate diminished neuromodulatory drive, but rather reflect an alteration in inhibitory control that attenuates PIC expression. This interpretation provides a plausible mechanistic explanation for the apparent dissociation, whereby brace height remains preserved (suggesting intact neuromodulatory facilitation) while Δ*F* is reduced (reflecting increased inhibitory downregulation of PICs). However, it is important to note that brace height has been primarily adopted (and validated) in within‐subject designs. To our knowledge, no prior cross‐sectional studies have detected between‐group differences in brace height; therefore, the sensitivity of this metric for detecting between‐group differences remains unknown. Thus, the lack of a group effect on brace height in our study does not completely rule out reduced neuromodulatory drive as a contributing mechanism to the Δ*F* differences observed in sarcopenia. On the other hand, the elevated Δ*F* in masters athletes in our study may stem from adaptive changes in the opposite direction. Long‐term exercise exerts neuroprotective effects, potentially preserving central serotonergic/noradrenergic pathways and receptor sensitivity, thereby sustaining stronger neuromodulatory drive during high‐intensity contractions. Athletes also show lower chronic oxidative stress and inflammation [36], potentially shielding high‐threshold motoneurons from degeneration. Notably, our exploratory analyses indicate that Δ*F* values were higher in power‐trained compared with endurance‐trained athletes. The repeated high‐intensity, rapid contractions characteristic of power‐type exercise, which consistently recruit high‐threshold motor units, may preferentially promote the preservation of PIC expression under comparable testing conditions (i.e., higher intensity contractions). Collectively, these adaptations could preserve intrinsic motoneuron properties and enhance PIC activation when motor output is most demanding, as reflected by our results. Elucidating the cellular and neuromodulatory mechanisms that impair or preserve PICs across ageing phenotypes remains an important avenue for future research.

### 4.3 Clinical Relevance and Applications

Our findings suggest that effective diagnosis and management of sarcopenia should consider possible neural impairments. Motor output assessments may be effective in sarcopenia case‐finding, and novel therapeutic approaches could target the neuromotor system. Traditional resistance training will likely remain one of the best tools for this, as it can induce neural adaptations (e.g., increased firing rates and PIC amplitude) [18, 19]. Indeed, the superior intrinsic motoneuron excitability in our athletes likely underscores how long‐term high‐intensity exercise can partially preserve neural function. Additionally, there may be a role for novel therapies aimed at enhancing motoneuron excitability and pharmacological approaches that enhance serotonergic or noradrenergic drive onto motoneurons that should be studied as adjuncts for individuals who cannot sufficiently engage the neuromuscular system through exercise [20, 37]. Such approaches would need careful testing in future studies, but they are conceptually supported by our finding that PICs are depressed in this group. In summary, our data may advocate for a more holistic clinical approach to sarcopenia where it is recognized as a neuromuscular condition, rather than purely as a muscle disease.

### 4.4 Strengths and Limitations

The use of high‐density surface EMG and motor unit decomposition allowed us to analyze nearly 5000 motor unit firings, providing a large and representative sample of motor unit behaviour in each group of older adults. Another important strength of this study is the clear contrast in physical function between groups, which maximized our ability to detect neural differences and relate them to functional outcomes, which might not have been apparent in previous studies [38, 39]. Despite these strengths, several limitations must be acknowledged. The sample size of the sarcopenic group was relatively small (*n* = 12), reflecting the difficulty of recruiting older adults with confirmed sarcopenia that are not taking medications directly affecting the monoaminergic system, such as antidepressants, and that are willing to perform physical function tests^S11,S12^. While the main outcome (i.e., ∆F) and several characterization variables displayed statistically significant differences between sarcopenic and the other groups, the modest sample size increases the risk of a type II error, limiting our ability to detect real differences that might exist for the variables displaying no statistical difference (e.g., total body SMM and ASMM). Moreover, our modest sample means that some caution is needed in generalizing the magnitude of excitability impairment to all sarcopenic populations (especially if using different sarcopenia cutoff points). Age differences between groups represent a limitation of this study. While sarcopenic individuals were about a decade older than masters athletes, these groups reflect distinct functional phenotypes rather than merely different age brackets. Age is a known contributor to neuromuscular decline, and its influence cannot be entirely disentangled from the effects of physical inactivity or lifelong training. Although the exploratory analysis indicated that the inclusion of age as a covariate had not marked impact on the main effects and interaction terms, and the estimated means remained consistent, the CIs were less precise for some of the contrasts (Supplementary Material S2). Thus, while age‐related effects are typically more pronounced when comparing younger and older adults, we acknowledge that residual confounding by age remains possible. Future studies with age‐matched cohorts will be important to more precisely isolate the respective contributions of aging and physical activity history to intrinsic motoneuron function. The cross‐sectional design also precludes any conclusions about causality. We cannot definitively say whether reduced motoneuron excitability leads to poor function or if individuals with severe functional impairments/sarcopenia develop reduced excitability as a secondary consequence. Furthermore, we recorded from only the tibialis anterior, which is a muscle that seems to display reductions of discharge rates with aging [10] but does not have the same degree of central activation impairment as other muscles [40]; thus, caution is advised in extrapolating our results to other muscle groups. We interpreted Δ*F* as a proxy for PIC based on strong theoretical and experimental justification [14], but we acknowledge it is not a direct measure. Physical activity was assessed via the IPAQ‐E questionnaire, which may be subject to recall and social‐desirability biases. However, it has been previously validated against accelerometer measurements, supporting its utility for estimating physical activity levels in older adults^S4^.

## 5 Conclusions

This study provides evidence that impaired intrinsic motoneuron excitability contributes to sarcopenia‐related physical decline. Older adults with sarcopenia included in this study exhibited reduced Δ*F* and lacked the capacity to scale excitability with increasing effort, features not observed in nonsarcopenic controls or masters athletes. In contrast, masters athletes preserved the ability to upregulate Δ*F* at higher intensities, which may help sustain physical function with age. Exploratory analyses indicated that power‐trained athletes exhibited the highest Δ*F* values, supporting the notion that power‐type exercise may preferentially promote the preservation of PIC expression. These findings indicate a neural dimension of sarcopenia and highlight intrinsic motoneuron excitability as a modifiable physiological feature that may be further investigated as both a potential biomarker and therapeutic target for mitigating age‐related neuromuscular decline.

## Conflicts of Interest

The authors declare no conflicts of interest.

## Supporting information


**Data S1:** Supporting information.

**Data S2:** Supporting information.

**Data S3:** Supporting information.

**Data S4:** Supporting information.
