# Supplementary material for: Intrinsic Motoneuron Excitability Differentiates Sarcopenic, Nonsarcopenic and Athletic Ageing Phenotypes
Source: J Cachexia Sarcopenia Muscle. 2025 Nov 25;16(6):e70126. doi: 10.1002/jcsm.70126 (PMC12647921; doi:10.1002/jcsm.70126)
Supplement: Supplementary file 1 — Data S1: Supporting information. [file JCSM-16-e70126-s002.pdf]

Supplementary Table 1. Motor unit number per group, sex, and contraction intensities.

Intensity	Sarcopenia		Control		Athlete	
	Females	Males	Females	Males	Females	Males
All motor units						
i20%	16 (11, 30)	25 (22, 30)	29 (27, 34)	35 (32, 44)	39 (36, 40)	42 (34, 43)
i40%	15 (11, 28)	25 (16, 28)	27 (22, 31)	29 (27, 36)	31 (23, 40)	39 (35, 49)
i60%	14 (13, 20)	22 (22, 29)	23 (21, 27)	27 (22, 36)	21 (19, 33)	31 (26, 42)
Recruitment threshold 0-20%						
i20%	16 (11, 30)	25 (22, 30)	29 (27, 34)	35 (32, 44)	39 (36, 40)	42 (34, 43)
i40%	11 (9, 17)	19 (10, 20)	20 (16, 22)	24 (20, 29)	16 (13, 26)	30 (26, 34)
i60%	7 (6, 14)	12 (10, 17)	11 (7, 13)	16 (11, 21)	7 (5, 13)	20 (11, 22)
Recruitment threshold 20-40%						
i40%	3 (2, 7)	6 (5, 9)	8 (6, 9)	8 (5, 10)	12 (7, 13)	9 (7, 17)
i60%	4 (2, 5)	8 (8, 12)	10 (8, 12)	8 (8, 11)	11 (4, 11)	11 (9, 15)
Recruitment threshold 40-60%						
i60%	2 (1, 4)	3 (1, 5)	2 (2, 4)	2 (1, 3)	4 (2, 7)	4 (2, 6)

Data is presented as median (1st, 3rd quartiles). The number of motor units identified (i.e., motor unit yield) does not represent the total number of motor units in the muscle, nor is it necessarily proportional to it, across participants or groups. Motor unit decomposition from high-density electromyography can be influenced by several factors, including the distance between muscle and electrodes (e.g., due to subcutaneous fat thickness), the muscle's maximal anatomical cross-sectional area, and the spatial distribution of motor units [1].

Reference

1. Oliveira DS de, Casolo A, Balshaw TG, Maeo S, Lanza MB, Martin NRW *et al.* Neural decoding from surface high-density EMG signals: influence of anatomy and synchronization on the number of identified motor units. *J Neural Eng* 2022;**19**.
