# Supplementary material for: Intrinsic Motoneuron Excitability Differentiates Sarcopenic, Nonsarcopenic and Athletic Ageing Phenotypes
Source: J Cachexia Sarcopenia Muscle. 2025 Nov 25;16(6):e70126. doi: 10.1002/jcsm.70126 (PMC12647921; doi:10.1002/jcsm.70126)
Supplement: Supplementary file 2 — Data S2: Supporting information. [file JCSM-16-e70126-s001.pdf]

Supplementary material 2. Effects of age on ΔF

This document provides supplementary information on the inclusion of age as a covariate in the ΔF models.

In summary, incorporating age had no significant effect on ΔF and did not alter the main effects or interactions observed in the original models. The estimated marginal mean differences remain consistent with those presented in Figure 3; however, the confidence intervals were less precise, introducing uncertainty around three contrasts. Specifically, the contrasts between Sarcopenia and Athletes at i20% (rt0–20%), Athletes and Controls at i40% (rt20–40%), and Athletes and Controls at i60% (rt40–60%) now slightly cross zero. A summary of the statistical results with age included is provided below and visualised in Supplementary Figure 2.

In low-threshold units (rt0−20%), a significant group-by-intensity interaction was observed [β=−0.51 (−0.89,−0.14), t=−2.71]. No significant effect was observed for age [β=−0.22 (−0.48, 0.05), t=−1.61]. Sarcopenic individuals had significantly lower ΔF values compared to controls at i20%, i40%, and i60%, and to athletes at i40% and i60%. No significant differences were observed between athletes and controls. ΔF increased with contraction intensity in athletes and controls but remained unchanged in sarcopenia. In mid-threshold units (rt20–40%), main effects of group [β=−1.29 (−2.02,−0.55), t=−3.44] and intensity [β=0.43 (0.22,0.64), t=4.05] were observed without interaction. No significant effect was observed for age [β=−0.17 (−0.41, 0.06), t=−1.43]. ΔF was consistently lower in sarcopenic individuals compared to controls and athletes across all intensities, and ΔF increased from i40% to i60% regardless of group. In high-threshold units (rt40−60%), a significant group effect was observed at i60% [β=−2.18 (−3.26,−1.30), t=−3.95). No significant effect was observed for age [β=-0.06 (-0.41,0.30), t=-0.31]. Sarcopenia showed lower ΔF than controls and athletes. Sex was a significant predictor of ΔF across all bins. Male participants exhibited lower ΔF than females in motor units recruited from 0–20% [β=−0.80 (−1.28,−0.31), t=−3.23], 20–40% [β=−1.25 (−1.68,−0.82), t=−5.71], and 40–60% (β=−1.26 (−1.91,−0.61), t=−3.78].

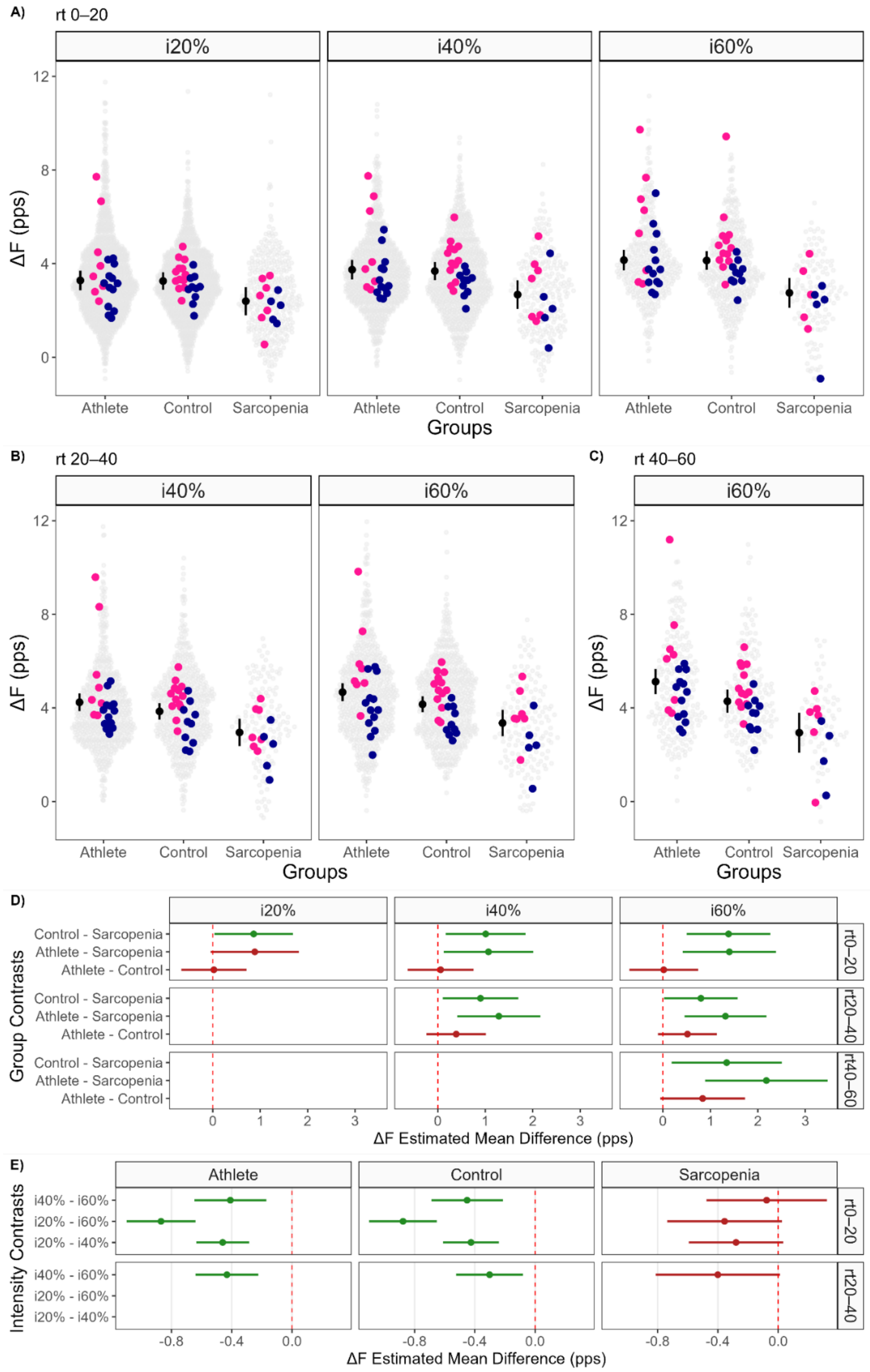

Supplementary Figure 2. Results from the ΔF model including age as a covariate. Panels A, B, and C present data stratified by recruitment threshold bins: 0–20%, 20–40%, and 40–60%, respectively. Each panel displays ΔF values across groups and contraction intensities. Black circles and lines represent estimated marginal means with their corresponding 95% confidence intervals. Average ΔF values for each female and male participant are shown as pink and blue circles, respectively. Transparent grey dots indicate individual motor unit data points. Panel D

shows group contrasts in ΔF at each contraction intensity, while Panel E illustrates ΔF contrasts across contraction intensities within each group. Circles and lines in Panels D and E represent estimated marginal mean differences and their 95% confidence intervals. Green indicates confidence intervals that do not cross zero (statistical significance), while red indicates non-significant findings. Note: Estimated marginal mean differences remain consistent with those presented in the original Figure 3; but with wider confidence intervals, introducing statistical uncertainty for some of the contrasts. More specifically, contrasts between Sarcopenia vs Athletes at i20% (rt0-20%), Athletes vs Controls at i40% (rt20-40%), and Athletes vs Controls at i60% (rt40-60%) are now slightly crossing zero.
