# Supplementary material for: Intrinsic Motoneuron Excitability Differentiates Sarcopenic, Nonsarcopenic and Athletic Ageing Phenotypes
Source: J Cachexia Sarcopenia Muscle. 2025 Nov 25;16(6):e70126. doi: 10.1002/jcsm.70126 (PMC12647921; doi:10.1002/jcsm.70126)
Supplement: Supplementary file 3 — Data S3: Supporting information. [file JCSM-16-e70126-s004.pdf]

Supplementary material 3. Endurance vs Power Athletes – Subgroup analysis

This supplementary material presents exploratory analyses comparing the endurance-type (n = 11) and power-type (n = 10) athlete subgroups on key characteristics, ΔF and Brace Height.

Participant characteristics

Compared to endurance athletes, power athletes demonstrated faster completion times on the 4SST and 5STS, as well as greater power output during the 5STS, and exhibited greater dorsiflexion peak torque when normalised by body mass. Handgrip strength and UGS were broadly comparable between groups. In terms of body composition, power athletes exhibited lower body fat percentage and higher phase angle, while total body SMM and ASMM were similar across groups.

Supplementary Table 2. Participants physical function and body composition per group and estimated marginal mean differences between groups.

	**Estimated marginal means (95%CI)**		**Estimated marginal mean differences (95%CI)**
**Physical function**	**Endurance**	**Power**	**Endurance – Power**
Handgrip strength, kg	33.5 (29.1, 37.8)	35.8 (31.2, 40.3)	-2.3 (-8.6, 3.9)
UGS, m·s^{-1}	1.36 (1.26, 1.45)	1.41 (1.31, 1.50)	-0.05 (-0.2, 0.08)
FGS, m·s^{-1}	2.07 (1.94, 2.20)	2.23 (2.09, 2.37)	-0.16 (-0.34, 0.03)
4SST, s	7.53 (7.20, 7.87)	6.79 (6.45, 7.14)	**0.74 (0.26, 1.21)**
TUG, s	5.96 (5.66, 6.25)	5.66 (5.35, 5.97)	0.30 (-0.13, 0.72)
5STS, s	9.91 (9.26, 10.57)	8.54 (7.87, 9.22)	**1.37 (0.44, 2.30)**
5STS, W·kg^{-1}	3.53 (3.23, 3.83)	4.03 (3.72, 4.34)	**-0.50 (-0.92, -0.08)**
Dorsiflexion peak torque, N·m	23.4 (20.2, 26.6)	27.4 (24.0, 30.7)	-4.0 (-8.6, 0.6)
Dorsiflexion peak torque/body mass, N·m·kg^{-1}	0.35 (0.30, 0.40)	0.45 (0.40, 0.49)	**-0.10 (-1.64, -0.03)**
**Body composition**			
Body mass, kg	66.3 (59.7, 73.0)	62.4 (55.5, 69.2)	4.0 (-5.5, 13.4)
Body fat %	22.2 (17.8, 26.6)	16.7 (12.1, 21.3)	5.5 (-0.8, 11.8)
Total body SMM, kg	23.6 (22.3, 24.9)	22.9 (21.5, 24.2)	0.8 (-1.1, 2.6)
ASMM, kg	14.8 (13.7, 15.8)	14.3 (13.2, 15.3)	0.5 (-1.0, 2.0)
Phase angle, degrees	5.1 (4.8, 5.5)	5.7 (5.3, 6.0)	**-0.5 (-1.0, -0.06)**

UGS, usual gait speed; FGS, fast gait speed; 4SST, four–square step test; TUG, timed up and go; 5STS, 5-times sit-to-stand; ASMM, appendicular skeletal muscle mass; SMM, skeletal muscle mass. Note: Bolded estimated marginal mean differences (95%CI) highlight confidence intervals not crossing zero, indicating statistical differences.

ΔF

In low-threshold units (rt0−20%), a significant group-by-intensity interaction was observed [β=0.50 (0.13, 0.87), t=2.63]. Power athletes displayed higher ΔF than endurance at i40% and i60%, but not at i20%. ΔF increased with contraction intensity in both subgroups (Supplementary Figure 2A).

In mid-threshold units (rt20–40%), main effects of group [β=1.00 (0.09,1.90), t=2.15] and intensity [β=0.33 (0.06,0.59), t=2.38] were observed without interaction. ΔF was lower in endurance compared to power athletes across all intensities, and ΔF increased from i40% to i60% regardless of group (Supplementary Figure 2B).

In high-threshold units (rt40−60%), no group effect was observed at i60% [β=0.99 (−0.26,2.22), t=1.56) (Supplementary Figure 2C).

Sex was a significant predictor of ΔF across all bins. Male participants exhibited lower ΔF than females in motor units recruited from 0–20% [β=−1.00 (−1.95,−0.05), t=−2.06], 20–40% [β=−1.51 (−2.42,−0.59), t=−3.23], and 40–60% (β=−1.44 (−2.71,−0.16), t=−2.21].

Brace Height

No group-by-intensity interaction or main effects were observed for rt0-20%, rt20-40%, and rt40-60% (Supplementary Figure 3). Sex differences emerged with males exhibiting lower brace heights in the rt0-20% [β=−3.68 (−6.31,−1.04), t=−3.68] and rt20–40% units [β=−5.60 (−9.62,−1.59), t=−2.73].

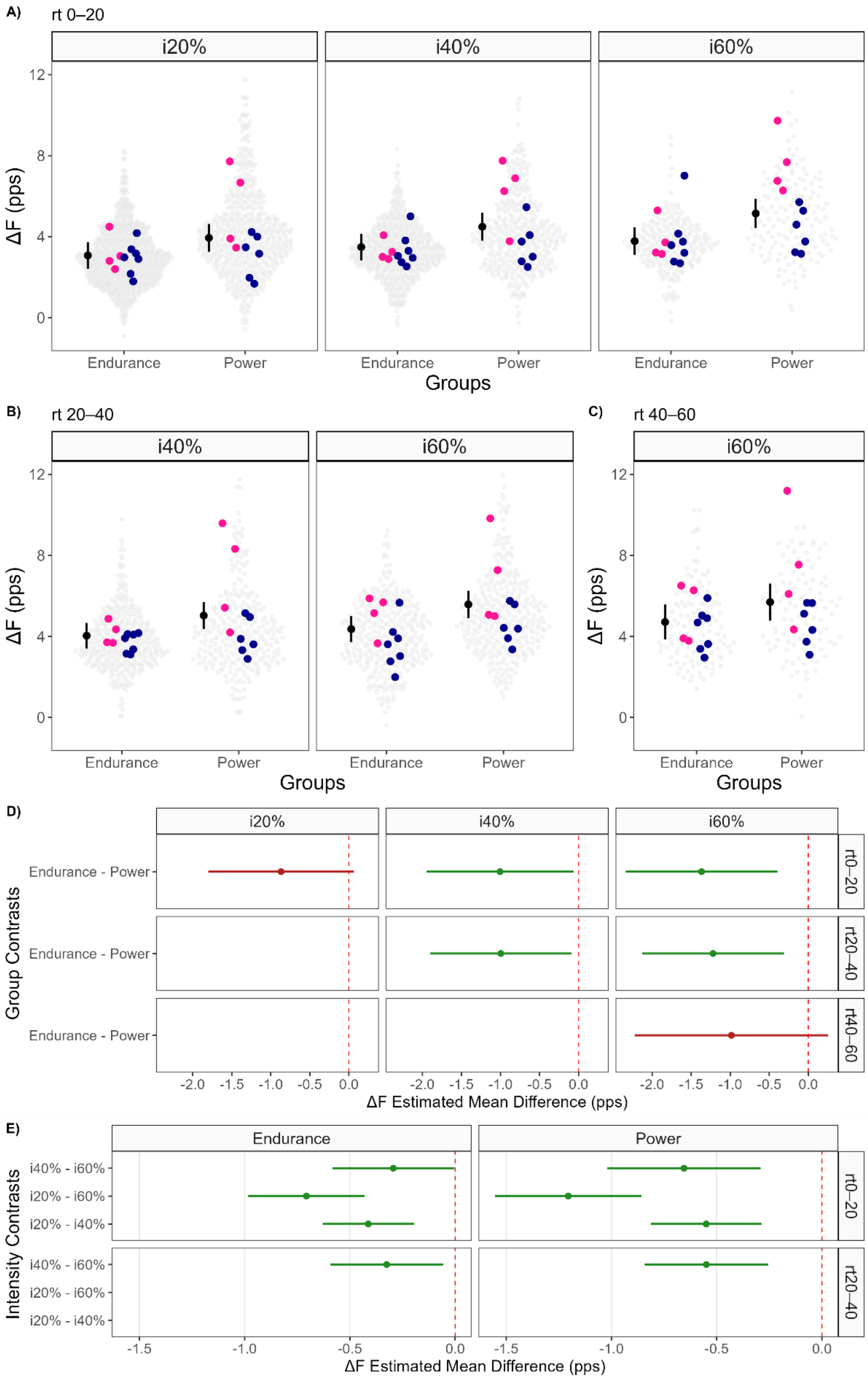

Supplementary Figure 2. Power vs endurance subgroup analysis - Intrinsic motor neurone excitability (ΔF). Panels A, B, and C present data stratified by recruitment threshold bins: 0–20%, 20–40%, and 40–60%, respectively. Each panel displays ΔF values across athletic subgroups (endurance and power) and contraction intensities (20%, 40%, and 60% of

participants' maximal force). Black circles and lines represent estimated marginal means with their corresponding 95% confidence intervals. Average ΔF values for each female and male participant are shown as pink and blue circles, respectively. Transparent grey dots indicate individual motor unit data points. Panel D shows group contrasts in ΔF at each contraction intensity, while Panel E illustrates ΔF contrasts across contraction intensities within each group. Circles and lines in Panels D and E represent estimated marginal mean differences and their 95% confidence intervals. Green indicates confidence intervals that do not cross zero (statistical significance), while red indicates non-significant findings.

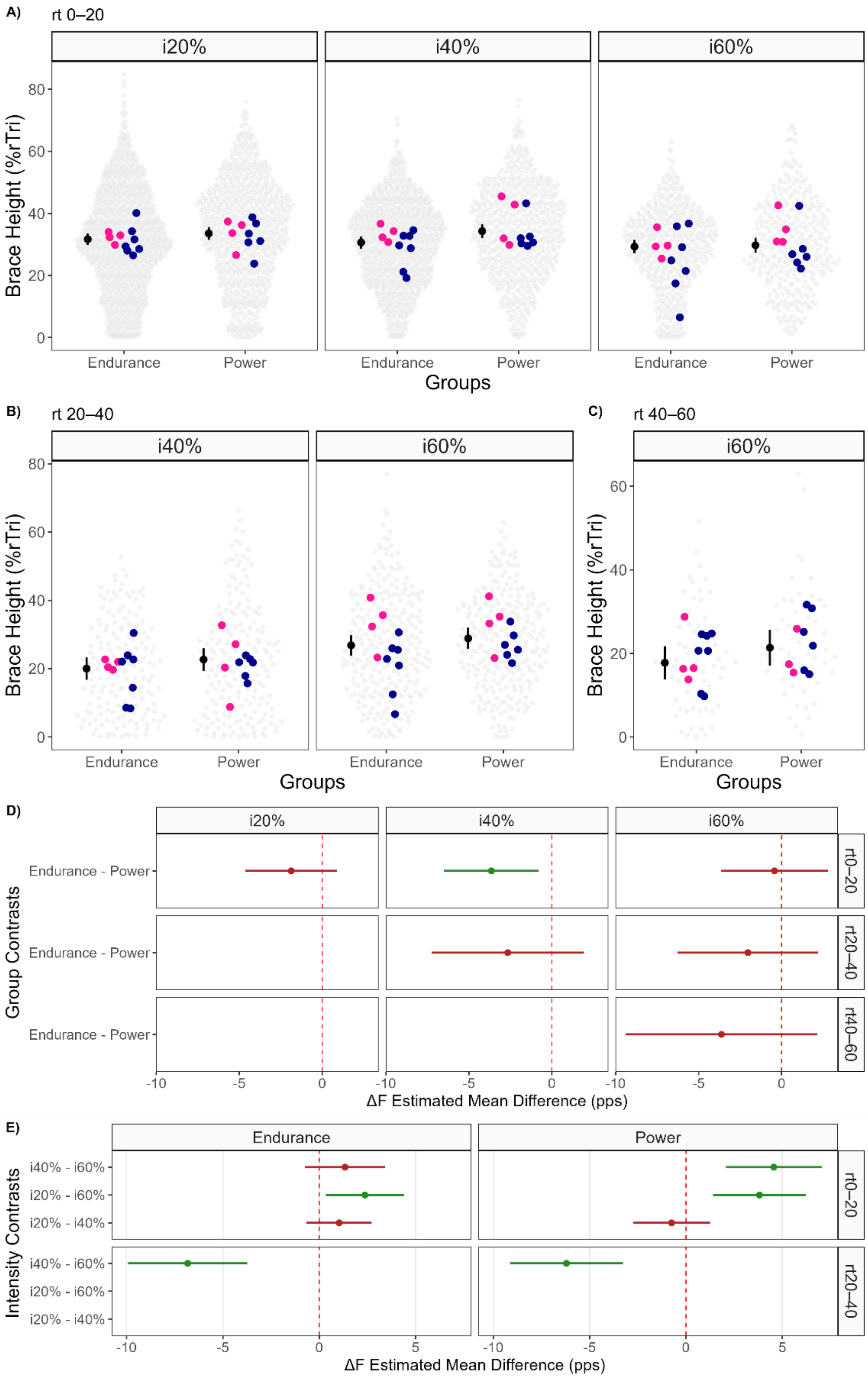

Supplementary Figure 3. Power vs endurance subgroup analysis – Brace Height. Panels A, B, and C present data stratified by recruitment threshold bins: 0–20%, 20–40%, and 40–60%, respectively. Each panel displays the normalised brace height values across athletic subgroups (endurance and power) and contraction intensities (20%, 40%, and 60% of participants' maximal force). Black circles and lines represent estimated marginal means with their corresponding 95% confidence intervals. Average normalised brace height values for each

female and male participant are shown as pink and blue circles, respectively. Transparent grey dots indicate individual motor unit data points. Panel D shows group contrasts in normalised brace height at each contraction intensity, while Panel E illustrates normalised brace height contrasts across contraction intensities within each group. Circles and lines in Panels D and E represent estimated marginal mean differences and their 95% confidence intervals. Green indicates confidence intervals that do not cross zero (statistical significance), while red indicates non-significant findings.
