# Supplementary material for: Intrinsic Motoneuron Excitability Differentiates Sarcopenic, Nonsarcopenic and Athletic Ageing Phenotypes
Source: J Cachexia Sarcopenia Muscle. 2025 Nov 25;16(6):e70126. doi: 10.1002/jcsm.70126 (PMC12647921; doi:10.1002/jcsm.70126)
Supplement: Supplementary file 4 — Data S4: Supporting information. [file JCSM-16-e70126-s003.pdf]

Supplementary References

S1. Katz S, Akpom CA. A measure of primary sociobiological functions. International Journal of Health Services 1976;6:493–508.

S2. Jette AM, Haley SM, Coster WJ, Kooyoomjian JT, Levenson S, Heeren T et al. Late Life Function and Disability Instrument: I. Development and Evaluation of the Disability Component. J Gerontol 2002;57:209–216.

S3. Cataldi D, Bennett JP, Wong MC, Quon BK, Liu YE, Kelly NN et al. Accuracy and precision of multiple body composition methods and associations with muscle strength in athletes of varying hydration: The Da Kine Study. Clinical Nutrition 2024;43:284–294.

S4. Hurtig-Wennlf A, Hagstrmer M, Olsson LA. The International Physical Activity Questionnaire modified for the elderly: Aspects of validity and feasibility. Public Health Nutr 2010;13:1847–1854.

S5. Hernández-Guillén D, Tolsada-Velasco C, Roig-Casasús S, Costa-Moreno E, Borja-De-Fuentes I, Blasco JM. Association ankle function and balance in community-dwelling older adults. PLoS One 2021;16.

S6. Perera CK, Gopalai AA, Ahmad SA, Gouwanda D. Muscles Affecting Minimum Toe Clearance. Front Public Health 2021;9.

S7. Afschrift M, van Deursen R, De Groote F, Jonkers I. Increased use of stepping strategy in response to medio-lateral perturbations in the elderly relates to altered reactive tibialis anterior activity. Gait Posture 2019;68:575–582.

S8. Fujimoto M, Hsu WL, Woollacott MH, Chou LS. Ankle dorsiflexor strength relates to the ability to restore balance during a backward support surface translation. Gait Posture 2013;38:812–817.

S9. Kemoun G, Thoumie P, Boisson D, Guieu JD. Ankle dorsiflexion delay can predict falls in the elderly. J Rehabil Med 2002;34:278–283.

S10. Lord SR, Murray SM, Chapman K, Munro B, Tiedemann A. Sit-to-Stand Performance Depends on Sensation, Speed, Balance, and Psychological Status in Addition to Strength in Older People. Journal of Gerontology: Medical Sciences 2002;57:539–543.

S11. Marzetti E, Cesari M, Calvani R, Msihid J, Tosato M, Rodriguez-Mañas L et al. The "Sarcopenia and Physical fRailty IN older people: multi-componenT Treatment strategies" (SPRINTT) randomized controlled trial: Case finding, screening and characteristics of eligible participants. Exp Gerontol 2018;113:48–57.

S12. Witham MD, Achison M, Aspray TJ, Avenell A, Band MM, Donnan PT et al. Recruitment strategies for sarcopenia trials: lessons from the LACE randomized controlled trial. JCSM Rapid Commun 2021;4:93–102.
